# Potential value of MicroRNA-21 as a biomarker for predicting the prognosis of patients with breast cancer: A protocol for meta-analysis and bioinformatics analysis

**DOI:** 10.1097/MD.0000000000025964

**Published:** 2021-06-04

**Authors:** Yunfeng Ding, Wanbo Wu, Zhihong Ma, Xia Shao, Ming Zhang, Zhanwei Wang

**Affiliations:** Department of Breast Surgery, Huzhou Central Hospital, Affiliated Central Hospital HuZhou University, Huzhou, Zhejiang Province, China.

**Keywords:** bioinformatics, breast cancer, meta-analysis, MicroRNA-21, protocol

## Abstract

**Background:**

The latest global cancer data from 2020 shows that breast cancer has replaced lung cancer as the number one cancer in the world. Searching for new biomarkers of breast cancer has important clinical significance for early diagnosis, prediction of prognosis, and targeted therapy. MicroRNA-21 (miRNA-21) can be used as a new molecular marker for early diagnosis, prognosis, and treatment of tumors. However, the expression of miRNA-21 in breast cancer and its prognosis are not clear. Therefore, this study conducted a meta-analysis to further clarify the relationship between the expression of miRNA-21 in breast cancer and prognosis. At the same time, we carried out bioinformatics analysis to further analyze the possible molecular mechanism of miRNA-21, so as to provide potential clinical indicators for the diagnosis, treatment, and prognosis of patients.

**Methods:**

PubMed, Medline, Embase, Web of Science, Wanfang, Chinese Biomedical Literature Database, Chinese National Knowledge Infrastructure, and other databases were used to retrieve the published relevant literatures. Include the eligible research, extract the survival data hazard ratios and 95% confidence intervals and other information. STATA16.0 software was used for meta-analysis. Download the miRNA data of breast cancer through the Cancer Genome Atlas (TCGA) database and Gene Expression Omnibus (GEO) database. The data extracted for independent sample *t* test and ROC curve was drawn. OncomiR plotted the survival curve of miRNA-21 on the prognosis of breast cancer. The target genes of miRNA-21 were predicted, and the Gene Ontology (GO) function and Kyoto Encyclopedia of Genes and Genomes (KEGG) pathway were analyzed. STRING database and Cytoscape construct protein-protein interaction (PPI) network to obtain Hub gene. The correlation between the expression level of Hub gene in breast cancer and the abundance of immune cell infiltration was analyzed by TIMER database and verified by Kaplan–Meien plotter database.

**Results:**

The results of this meta-analysis will be submitted to a peer-reviewed journal for publication.

**Conclusion:**

In this study, meta-analysis and bioinformatics analysis were used to further explore the prognosis, mechanism, and related pathways of miRNA-21 in breast cancer.

**Ethics and dissemination:**

The private information from individuals will not be published. This systematic review also should not damage participants’ rights. Ethical approval is not available. The results may be published in a peer-reviewed journal or disseminated in relevant conferences.

**OSF REGISTRATION NUMBER:**

DOI 10.17605/OSF.IO/R32A9.

## 1 Introduction

Breast cancer is one of the most common cancers in women around the world, which usually occurs in breast ducts and breast lobules, accounting for 22.90% of female cancers.^[1]^ At present, the diagnosis and treatment of breast cancer not only burden the physiology and psychology of patients, but also adversely affect the family and society.^[2]^ Therefore, in-depth understanding of the molecular pathogenesis of breast cancer, looking for biomarkers that are more sensitive to breast cancer, early detection, and treatment are of great significance to improve the overall survival time of patients with breast cancer.^[3]^

Micro-RNA (miRNA) is a kind of small molecular single-stranded non-coding RNA with a length of about 18–25 nucleotides.^[4,5]^ It can bind to the 3-non-coding region of the target gene RNA and function through RNA-mediated silencing complexes to prevent protein translation and expression or inhibit biological processes such as homologous mRNA degradation, thus exerting the function of tumor suppressor genes or tumor promoting genes.^[6]^ It has been found that miRNA plays an important role in apoptosis, proliferation, metabolism, and differentiation.^[7–9]^ This is closely related to the occurrence, proliferation, metastasis, diagnosis, treatment, and prognosis of the tumor.^[10–12]^

As the most frequently studied cancer-related miRNA, in clinic, the abnormal expression of MiRNA-21 is related to a variety of malignant tumors such as liver cancer, thyroid cancer, colorectal cancer, and so on.^[4,13–15]^ Studies have shown that in breast cancer patients, the up-regulated expression of miRNA-21 is closely related to tumor cell proliferation, invasion, and drug resistance.^[16,17]^

At present, the lack of sample size in the study of miRNA-21 expression and prognosis in patients with breast cancer leads to inconsistent results.^[18–23]^ The purpose of this study is to explore the prognostic significance of miRNA-21 expression in patients with breast cancer by meta-analysis, and to provide accurate medical basis for the clinical application of miRNA-21. In addition, we carried out bioinformatics studies to clarify the expression and significance of miRA-21, to explore its possible mechanism, and to provide experimental basis for the study of molecular markers of breast cancer.

## 2 Methods

### 2.1 Study registration

The protocol of the systematic review has been registered on Open Science Framework. The registration number is DOI 10.17605/OSF.IO/R32A9. This meta-analysis protocol is based on the Preferred Reporting Items for Systematic Reviews and Meta-analysis Protocols (PRISMA-P) Statement Guidelines.^[24]^

### 2.2 Data sources and search strategy

Comprehensive search of PubMed, Medline, Embase, Web of Science, Wanfang, Chinese Biomedical Literature Database, Chinese National Knowledge Infrastructure databases. The retrieval time is from the establishment to April 2021. Languages are limited to English and Chinese. Literature traceability, manual retrieval, and other methods are used to search the relevant literature. The search strategy for PubMed is shown in Table 1. According to the characteristics of each database, the retrieval strategy can be changed slightly.

**Table 1:** Search strategy in PubMed database.

Number	Search terms
#1	miRNA-21 [Title/Abstract]
#2	microRNA-21 [Title/Abstract]
#3	miR-21[Title/Abstract]
#4	hsa-miR-21[Title/Abstract]
#5	OR/1-4
#6	Breast Neoplasms [MeSH]
#7	Breast Cancer[Title/Abstract]
#8	Breast Tumors[Title/Abstract]
#9	Cancer of Breast[Title/Abstract]
#10	Cancer of the Breast[Title/Abstract]
#11	Human Mammary Carcinoma[Title/Abstract]
#12	Mammary Carcinoma, Human[Title/Abstract]
#13	Mammary Neoplasm, Human[Title/Abstract]
#14	Mammary Neoplasms, Human[Title/Abstract]
#15	Neoplasms, Breast[Title/Abstract]
#16	Tumors, Breast[Title/Abstract]
#17	Breast Neoplasm[Title/Abstract]
#18	Breast Tumor[Title/Abstract]
#19	Cancer, Breast[Title/Abstract]
#20	Carcinoma, Human Mammary[Title/Abstract]
#21	Carcinomas, Human Mammary[Title/Abstract]
#22	Human Mammary Carcinomas[Title/Abstract]
#23	Human Mammary Neoplasm[Title/Abstract]
#24	Human Mammary Neoplasms[Title/Abstract]
#25	Mammary Carcinomas, Human[Title/Abstract]
#26	Neoplasm, Breast[Title/Abstract]
#27	Neoplasm, Human Mammary[Title/Abstract]
#28	Neoplasms, Human Mammary[Title/Abstract]
#29	Tumor, Breast[Title/Abstract]
#30	OR/6–29
#31	survival[Title/Abstract]
#32	prognos^∗^[Title/Abstract]
#33	OR/31–32
#34	#5 AND #30 AND #33

### 2.3 Inclusion criteria for study selection

(1) Literature inclusion criteria: the study on breast cancer diagnosed by pathology; the expression level of miRNA-21 was detected in the study; the results showed the relationship between the expression level of miRNA-21 and the survival and prognosis of patients.
(2) Literature exclusion criteria: review, meta-analysis, conference summary, etc; repeated published articles or experimental data; articles that cannot be extracted from HR.

### 2.4 Data collection and analysis

#### 2.4.1 Selection of studies

**Figure 1:**
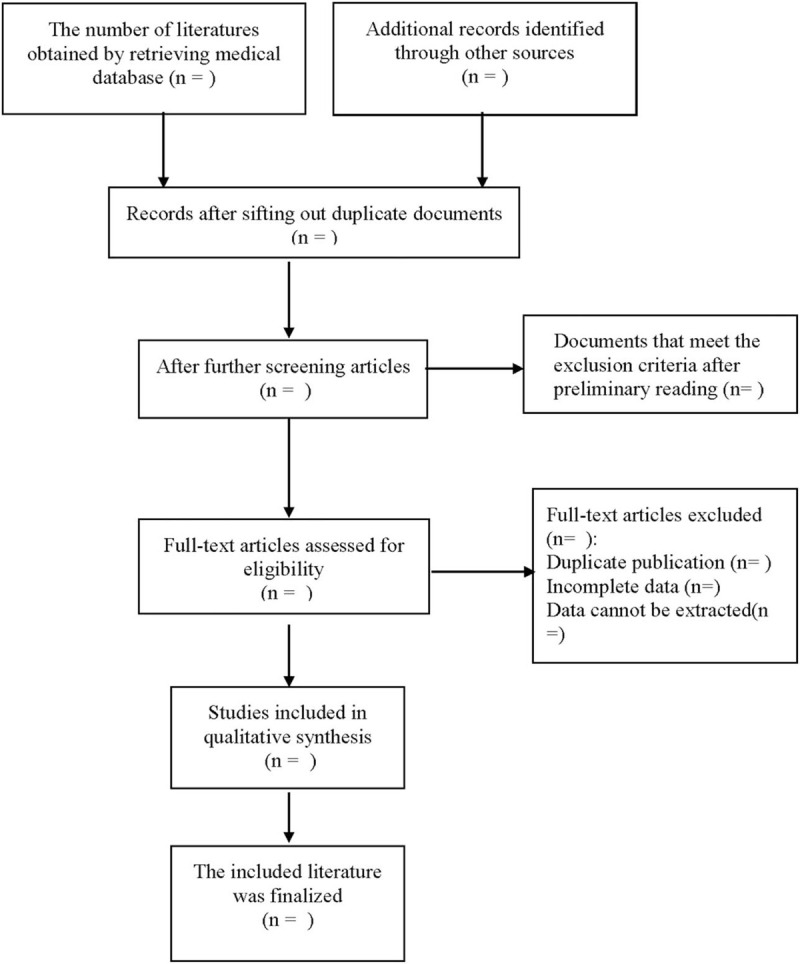
Flow diagram of study selection process.

The literature screening process is shown in Fig. 1. The titles and abstracts of the reading literature were screened by 2 researchers. The rest of the literature will be rescreened by reading the full text. Also, references of relevant literature are manually searched for screening to exclude those that do not meet the requirements. Disagreements were judged by discussion or by a third party when they arose.

#### 2.4.2 Data extraction and management

The extracted data include: the first author, the year of publication, country or region, race, sample number, sample type, tumor TNM stage, detection method, research type, cutoff value of miRNA-21 expression, adjuvant therapy, 95% confidence interval (95% CI), HR, and so on. If there is no survival outcome of HR, 95% CI, etc, use the method described by Tierney et al^[25]^ to extract effective information according to the data in the article.

### 2.5 Assessment of quality in included studies

Literature quality was evaluated based on the Newcastle-Ottawa Quality Assessment Scale (NOS).^[26]^ The total score is 9 points, and NOS score ≥7 is classified as a high-quality study.^[27]^

### 2.6 Measures of prognosis

OS and DFS will be taken as prognostic outcomes. The results will be expressed as HRs, with 95% CIs.

### 2.7 Management of missing data

If there exists insufficient or missing data in the literature, we would only analyze the currently available data and discuss its potential value.

### 2.8 Statistical analysis

STATA 16.0 (STATA Corporation, College Station, TX) was used for this meta-analysis. HR and its 95% CIs were used to evaluate the relationship between miRNA-21 expression and clinical prognosis in patients with breast cancer. The chi-squared test and *I*^*2*^ values were carried out to assess the heterogeneity among the pooled analysis. If *P* < .1 or *I*^*2*^ > 50% indicates that there is significant heterogeneity between studies, the random effect model is used to deal with the survival data, otherwise the fixed effect model would be used.

### 2.9 Additional analysis

#### 2.9.1 Subgroup analysis

We will conduct a subgroup analysis based on cutoff value of miRNA-21 expression, ethnicity, and survival data sources.

#### 2.9.2 Sensitivity analysis

The sensitivity analysis was carried out by using the method of excluding the study one by one.

#### 2.9.3 Publication bias

If no <10 articles are included, we will use funnel chart to test publication bias.^[28,29]^

### 2.10 Bioinformatics analysis

#### 2.10.1 Expression of miRNA-21 in breast cancer

MiRNA-21 expression data of breast cancer and normal tissues were downloaded from GEO database and TCGA database. The mean and standard deviation of miRNA-21 expression in breast cancer tissues and normal controls were calculated. Independent sample *t* test was used to draw ROC curve to test the diagnostic value of miRNA-21.

#### 2.10.2 Expression of miRNA-21 and prognosis of breast cancer

The OncomiR database (http://www.oncomir.org/) was used to draw the survival curve between the expression of miRNA-21 and the prognosis of breast cancer.

#### 2.10.3 Target gene prediction

The target genes of miRNA-21 were predicted and analyzed by using the integrated softwares of DIANAmT, MicroInspector, miRanda, MirTarget2, miTarget, NBmiRTar, PicTar, PITA, RNA22, RNAhybrid, and Targetscan in miRecords website (http://c1.accurascience.com/miRecords/). At least 3 target genes supported by the software were selected for signal pathway analysis.

#### 2.10.4 GO and KEGG analysis

DAVID database (https://david.ncifcrf.gov/) was used for Gene Ontology (GO) and Kyoto Encyclopedia of Genes and Genomes (KEGG) enrichment analysis.

#### 2.10.5 Hub gene acquisition

String database (https://www.string-db.org/) and Cytoscape constructed PPI network to obtain Hub genes.

#### 2.10.6 The relationship between Hub gene expression and prognosis of gastric cancer

The mRNA expression level of Hub gene in breast cancer was analyzed by Gene Expression Profiling Interactive Analysis (GEPIA). Survival curves of Hub genes and breast cancer prognosis were plotted by Kaplan–Meier Plotter database (http://www.kmplot.com/analysis/index.php?p=service&cancer=gastric).

#### 2.10.7 Hub gene expression and immune infiltrating cells

TIMER database (http://cistrome.dfci.harvard.edu/TIMER/) was used to analyze the relationship between Hub gene expression and immune cell infiltration in breast cancer, which included B cells, CD4+ T cells, CD8+ T cells, neutrophils, macrophages, and dendritic cells.

#### 2.10.8 Relationship between immune infiltration and prognosis of patients with breast cancer

Kaplan–Meier Plotter database was used to analyze the effect of immune cell infiltration on the prognosis of breast cancer patients with high and low expression of Hub gene.

### 2.11 Ethics

Our research data were derived from published literatures, because there were no patient recruitment and personal information collection. Therefore, ethical approval was not needed.

## 3 Discussion

As a kind of miRNA, the expression level of MiRNA-21 was first reported to be related to cancer in 2005.^[30]^ In recent years, studies have shown that the abnormal expression of miRNA-21 is closely related to the occurrence and development of many kinds of malignant tumors.^[15]^ MiRNA-21 is an oncogene, which not only plays a role in the proliferation of breast cancer, but also relates to tumor invasion, metastasis, and apoptosis. It reduces the expression of tumor suppressor genes to promote the occurrence and development of tumors. At present, many target genes of miRNA-21 have been found, including programmed death protein 4, tropomyosin 1, homologous lost phosphatase tensin on chromosome 10, Fas ligand, tissue inhibitor of metalloproteinase-3, tumor-associated protein 63, and so on.^[10,31–33]^ MiRNA-21 participates in a series of life activities of tumor by regulating these target genes.

MiRNA-21 is a serotype marker, which can be released into the blood from tissue cells when breast cancer occurs, and rises in the early stage of breast cancer. It can sensitively and specifically reflect the occurrence and development of breast cancer. In this study, meta-analysis was used to further analyze the relationship between the expression of miRNA-21 and the prognosis of patients with breast cancer, so as to determine that miRNA-21 can be used as a potential clinical indicator. By means of bioinformatics, we can obtain the regulatory targets and signal pathways of miRNA-21. In addition, by analyzing the correlation between Hub gene expression and immune infiltrating cells, we can provide experimental basis for how miRNA-21 target genes affect the biological mechanism of tumor microenvironment regulating the occurrence and development of breast cancer.

## Author contributions

**Data curation:** Yunfeng Ding, Wanbo Wu.

**Funding acquisition:** Zhanwei Wang.

**Investigation:** Ming Zhang, Zhanwei Wang.

**Methodology:** Xia Shao.

**Resources:** Zhihong Ma.

**Software:** Xia Shao.

**Supervision:** Zhihong Ma, Ming Zhang, Xia Shao, Yunfeng Ding, and Wanbo Wu.

**Validation:** Zhihong Ma and Zhanwei Wang.

**Visualization and software:** Zhihong Ma and Xia Shao.

**Writing – original draft:** Yunfeng Ding, Wanbo Wu.

**Writing – review & editing:** Yunfeng Ding and Zhanwei Wang.
